# Chemokine modulation in microscopic and submicroscopic *Plasmodium falciparum* malaria infection in women at delivery in Yaoundé, Cameroon

**DOI:** 10.1371/journal.pone.0280615

**Published:** 2023-01-23

**Authors:** Rosette Megnekou, Chris Marco Mbianda Nana, Jean Claude Djontu, Bernard Marie Zambo Bitye, Benderli Christine Nana, Berenice Kenfack Tekougang Zangue, Christiane Josiane Donkeu, Estelle Essangui, Rodrigue Mbea Salawiss, Reine Ndeumou Medouen Seumko’o, Lawrence Ayong, Rose Gana Fomban Leke

**Affiliations:** 1 Department of Animal Biology and Physiology of the Faculty of Science, University of Yaoundé I, Yaoundé, Cameroon; 2 The Immunology Laboratory of the Biotechnology Center, University of Yaoundé I, Yaoundé, Cameroon; 3 Malaria Research Unit, Centre Pasteur du Cameroun, Yaoundé, Cameroon; Ehime Daigaku, JAPAN

## Abstract

In pregnancy-associated malaria, chemokines such as CXCL-4, CXCL-13, CXCL-16, and CCL-24 play critical roles in leucocyte trafficking to tissue sites in the infected placenta where inflammatory reactions are active. However, how plasma levels of these chemokines associate with *Plasmodium falciparum* placental malaria and pregnancy outcomes remains not well understood. The present study analyzed the plasma levels of CXCL-4, CXCL-13, CXCL-16, and CCL-24 chemokines in matched peripheral, placental and cord blood in relation with placental malaria (PM), and with submicroscopic parasitaemia. This was a retrospective case-control study (1:3 ratio) involving samples from 134 women (34 PM+ and 100 PM-) enrolled at delivery at the Marie Reine Health Center in Yaoundé, Cameroon between June 2013 and October 2018. Samples were collected just after delivery and used to diagnose microscopic and submicroscopic *Plasmodium falciparum* infections. Submicroscopic infections were detected by reverse transcription LAMP whereas chemokine levels were determined by Magnetic Luminex Screening Assay. Overall, PM was associated with increased plasma levels of CXCL-13 and CXCL-16 and low levels of CXCL-4 and CCL-24 in both peripheral and placental blood (0.0002 ≤ p ≤ 0.042). Similarly, CCL-24 levels in peripheral and placental blood samples were significantly lower in submicroscopically infected women compared to healthy controls (p = 0.04 and 0.02, respectively). Maternal hemoglobin levels increased with peripheral plasma levels of CXCL-4 (p = 0.005), CXCL-16 (p = 0.03), and CCL-24 (p = 0.002) while birth weight was lower for babies born from women with high levels of peripheral CXCL-13 (p = 0.0006) and low levels of cord CXCL-4 and CCL-24 (p = 0.02 and 0.08, respectively). Together the data suggest that low levels of CXCL-4 and CCL-24 coupled with high plasma levels of CXCL-13 and for a lesser extend CXCL-16 represent signatures of PM in the study population. These findings are relevant for understanding the immunopathogenesis of PM and developing new therapeutic or preventive strategies against severe PM outcomes.

## Introduction

*Plasmodium falciparum* malaria remains a leading cause of mortality and morbidity in new-borns, particularly in sub-Saharan Africa where about 40 million women become pregnant each year [1]. In 2021, about 13.3 million women in sub-Saharan Africa were exposed to *P*. *falciparum* infection during pregnancy, resulting in 961 000 babies with low birth weights [1]. The main determinants of this include the sequestration of parasitized erythrocytes in the placental tissue causing placental malaria (PM) [2] and the subsequently strong inflammatory responses resulting from alteration of pro and anti-inflammatory cytokine/chemokine balance in the feto-maternal interface [3–6]. This condition might cause the intervillositis that alters efficient blood flow across the placenta, leading to poor pregnancy outcomes including fetal anemia, preterm delivery, and low birth weight [5–7]. Increasing evidence from human studies revealed the presence of host leucocytes at the site of parasite sequestration in the placental tissue [8]. These findings indicate that in addition to cytokines secretion, leucocytes might also contribute to the disease severity by migrating to placental tissue thereby exacerbating organ-specific inflammation.

Chemokines are best known for their ability to stimulate the migration of cells, mostly white blood cells [9]. As the first mobilizers of host response, chemokines play crucial role in the development and homeostasis of the immune system, and are involved in all protective immune and inflammatory responses [9]. The chemokine CXCL-4 also called Platelet Factor 4 (PF-4) is released by activated platelet α-granules [10]. This chemokine together with CXCL-13 and CXCL-16 have been shown to be involved in the implantation and embryonic development early during pregnancy [11, 12]. Moreover, data from previous reports suggest that *Plasmodium-*infected erythrocytes increase plasma levels of Th1 related chemokines including CXCL-13 and CXCL-16, leading to the formation of an ectopic germinal center which serves as a tertiary lymphoid organ in infected tissues [3, 11]; and such a Th1 immune response bias is generally associated with low birth weight [4, 13].

The diagnosis of PM infection remains a major challenge as 20–25% of PM infections are misdiagnosed in women from sub-Saharan Africa when using the microscopic examination of Giemsa-stained peripheral thick blood smears [14, 15], the WHO gold standard method. High sensitive methods such as nested-PCR, LAMP and RT-LAMP have been developed for the detection of low parasite density related infections including submicroscopic malaria infection [16, 17]. However, these techniques are very expensive, time consuming, require highly trained personnel, and cannot provide the information on the inflammatory status of the placental tissue. Therefore, the identification of molecular correlates of PM and submicroscopic infections in particular, as well as markers of protection remain invaluable.

Similar to microscopic parasitemia, submicroscopic malaria parasiteamia has been associated with maternal anemia, low birth weight [16] and alteration of the cytokine profile [18]. According to previous reports, submicroscopic *Plasmodium falciparum* infections during pregnancy are able to stimulate immune response mediators such as IFN-γ, TNF-α, and IP-10 altering their expression patterns [18, 19]. These evidences attracted substantial interest in identifying among chemokines useful biomarkers of *P*. *falciparum* PM. Most of the previous cytokines /chemokines identified as *Plasmodium* related markers are involved in different pathophysiological pathways, and thus, lack specificity to malaria infection. In addition, the majority of these cytokines/chemokines have not yet been analysed in relation with submicroscopic malaria parasitaemia, although high proportion of malaria infected pregnant women from sub-Saharan Africa are known to be asymptomatic, and bearing mostly submicroscopic parasitaemia [20, 21]. The present study therefore aimed to analyze the plasma levels of the pregnancy-associated chemokines CXCL-4, CXCL-13, CXCL-16, and CCL-24 in matched peripheral, placental, and cord blood, in relation with PM including submicroscopic parasitaemia and pregnancy outcomes, in women reporting for delivery in Yaoundé, Cameroon. This study might provide leads that should help to identify host molecules likely to be used as markers of PM or as correlates of protection.

## Materials and methods

### Ethics statement

Participation in the study was voluntary with written Informed Consent or Assent from each participant or their guardian prior to recruitment. The study protocol was reviewed and approved by the National Ethics Committee of Cameroon (Ethical Clearances N° 2013/02/ No 029/L/CNERSH/SP and N°2018/07/1067/CE/CNERSH/SP). Administrative Authorizations were obtained from the Ministry of Public Health of Cameroon (N° D30-392 AAR/MINSANTE/SG/DROS/ CRC/ CEA1) and from local health authorities. The study was performed following the guidelines and regulations of human clinical research as recommended by the Ministry of Public Health of Cameroon. Malaria rapid diagnostic test (RDT) was performed for each woman at the time of enrolment and positive results were reported to the physician for prescription of treatment according to the national guidelines.

### Study site and population

Participant enrolment took place at the Marie Reine Health Center of Etoudi in Yaoundé, Cameroon. It was a retrospective case control study (1:3 ratio), involving an initial study undertaken between 2013 and 2018, where peripheral, placental and cord blood samples were collected from 134 women (34 placental malaria positive and 100 negative) enrolled at delivery. In addition, 134 pieces of placenta from the same women were collected immediately following delivery. Malaria transmission in this site is perennial, with 2 wet and 2 dry seasons. Entomological inoculation rates were estimated at 32 infectious bites per person per year in this peri-urban area of Yaoundé [22]. Information on the mother’s health, estimated length of pregnancy, parity, age, use of antimalarial drugs, HIV status, and baby weight were recorded in a standard questionnaire. Birth weights lower than 2.5 kg were considered as low this is the standard definition of low birthweight as per the World Health Organization’s guidelines [23]. Peripheral, placental and cord blood samples were aseptically collected in EDTA tubes. A portion of each sample was used to prepare smears for microscopic diagnostic of malaria infection and detection of submicroscopic malaria infection by RT-LAMP method. The remaining sample was centrifuged and the plasma collected and stored at -80°C for chemokine measurements. Placental tissues were also collected and used to prepare impression smears and for histology.

### Diagnosis of placental malaria and determination of hemoglobin levels

For the microscopy-based diagnosis of malaria, maternal peripheral, placental and cord blood were used to prepare thick and thin blood smears while placenta tissue was used to prepare impression smears. Slides were stained using Giemsa and examined by two skilled microscopists for the presence of malaria parasites. *P*. *falciparum* infection was further established using the rapid diagnostic test, malaria Carestart^™^ HRP2 (Pf) (Access Bio Inc, NJ, USA). Placental tissue sections were fixed in buffered formalin, embedded, stained with haematoxylin-eosin, and examined for the presence of parasites and malaria pigments. A woman was considered placental *P*. *falciparum* positive if infected erythrocytes were detected in impression smears of placenta tissue, placental blood smear or in histological sections. The presence of malaria pigment in placental tissue was declared when detecting pigment within monocytes from placental impression smear and/or in intervillous spaces of placental histological sections. Acute placental *P*. *falciparum* infection was characterized by the presence of infected erythrocytes in impression smears of placenta tissue, placental blood smear or in histological sections with no malaria pigment in the placental tissue. Hemoglobin (Hb) levels in maternal blood and neonate cord blood were determined using a Coulter counter (URIT-3300, Europe). A woman was considered anemic if Hb < 11 g/dL, whereas new born was considered anemic if Hb < 12,5 g/dL.

### Sample selection

In the present study, peripheral, placental and cord sera from 34 PM positive women (PM+) and 100 controls (PM-) were used. The 34 PM positive sera were selected based on the availability of the following informations: women’s age, gravidity and malarial status at delivery based on placental tissue impression smears. Then, peripheral, placental and cord sera from 100 PM-negative women of age 16 to 44 years and gravidity 1 to 9 matched to the selected PM+ women to avoid bias. That is, for each case (PM+), three controls of similar age or gravidity were enrolled for comparative analyses.

### Diagnosis of submicroscopic malaria infection

The detection of *P*. *falciparum* submicroscopic malaria infection was performed using the RT-LAMP technique as previously described [17]. Briefly, the blood samples were diluted (1:50) in a lysis buffer comprising 10 mM Tris-buffered saline, pH 7.4, 0.2% Triton X-100, and 0.1% bovine serum albumin and kept at room temperature for 2 minutes. The lysate (2.5 uL) was immediately added to a reaction mix comprising 2 μl of pre-mixed primers forward (F3) and backward (B3) primers, forward-inner (FIP) and backward-inner (BIP) primers, and loop forward (LF) and loop backward (LB) primers), 15 μL of reconstituted enzyme (ISO-DR001, OptiGene, UK) and 5.5 μl of DEPC-treated water [24]. The RT-LAMP reaction was then performed at 68°C for 45 minutes using a Genie II real-time amplifier. An RT-LAMP inactivation/annealing step of 98–70°C with ramp at 0.1°C per minute was included, and the derived melting curves used to verify the reaction specificity. Samples were considered positive if resulting in an amplification peak with a characteristic annealing temperature of ~86.5°C [24]. Submicroscopic infection was characterized by a positive peripheral blood RT-LAMP result and a negative placental tissue impression smear result with absence of parasite in the placental histological sections.

### Measurement of chemokines

Plasma levels of CXCL-4, CXCL-13, CXCL-16, and CCL-24 were measured by magnetic luminex screening assay, using Human Premixed Multi-Analyte Kit (R&D Systems, Inc. Minneapolis, MN, USA). Human, magnetic, premixed, microparticle cocktail of antibodies specific to CXCL-4, CXCL-13, CXCL-16, and CCL-24 was used to simultaneously screen maternal peripheral, placental and neonate cord plasma. The assay was carried out according to manufacturer’s instructions. Briefly, plates were incubated on the horizontal shaker with 50 μL/well of different diluted samples and concentration of standard chemokines provided alongside with 50 μL of human, magnetic, premixed, microparticle cocktail with antibodies specific for each chemokine. After washing using magnetic plate separator (Luminex, Austin, TX, USA, Cat# CN-0269-01), plates were incubated with 50 μL/well of human premixed biotin-antibodies cocktail specific for each chemokine. Antibody chemokine complexes were revealed using Streptavidin-PE. Plates were read using a Luminex MAGPix Analyzer (XMAP Technology, SN, USA) and results expressed as median fluorescence intensity (MFI). A standard curve was generated for each chemokine to convert MFI into corresponding chemokines relative concentrations.

### Statistical analysis

The GraphPad Prism 6.0.1 software was used for statistical analyses. Results were reported as means with standard deviation or medians with interquartile ranges. Mann–Whitney rank sum test or Kruskal wallis test was used to evaluate inter-group differences in the levels of chemokines. Proportions were compared using Fisher’s exact test. P values <0.05 were considered statistically significant.

## Results

### Study population

The baseline characteristics of women involved in this study are summarized on the Table 1. Samples from 34 placental malaria positive (PM+) women (16 women with PM chronic infection, 18 women with PM acute infection) and 100 placental malaria negative (PM-) women (43 women with peripheral blood submicroscopic infection, 57 healthy controls) were used for the study. The mean age (25.4 years) and gravidity (2.2) of PM+ women were not significantly different from those of PM- women (26.3 years and 2.2, p = 0.43 and 0.96, respectively). As shown in Table 1, maternal hemoglobin levels in the present study were significantly lower in PM+ women (10.85 g/dL for PM chronic infection, 11.00 g/dL for PM acute infection) compared to PM-women (12.35 g/dL for PM- with peripheral blood submicroscopic infection, 12.60 dL for healthy controls) (p < 0.0001), and the prevalence of maternal anemia was higher in PM+ (50% for PM chronic infection, 50% for PM acute infection) than in PM- (19.0% for PM- with peripheral blood submicroscopic infection, 12.3% for healthy controls) (p = 0.0006). The mean birth weight of babies born from women with infected placenta (2,988 g and 3,122 g for PM chronic and PM acute infections respectively) was lower compared to that of babies born from PM- women (3,433 g and 3,239 g for PM-with peripheral blood submicroscopic infection and healthy controls respectively) (p = 0.006), although the percentage of baby with low birth weight was similar in PM+ (5.6%) and PM-women (5.0%). The pregnancy period as well as the preterm delivery prevalence were not significantly different across the different groups, and the identified low birth weight cases appear not related to premature delivery. The hemoglobin levels tended to be lower in neonates born from PM+ women (14.80 g/dL for neonate from PM chronic infection, 14.60 g/dL for neonate from PM acute infection) compared to those of neonate from PM-women (15.00 g/dL for PM- with peripheral blood submicroscopic infection, 15.30 dL for healthy controls), although not significant (p = 0.66). However, the prevalence of fetal anemia was higher in PM+ (20% for neonate from PM chronic infection, 22.2% for neonate from PM acute infection) than in PM- (9.3% for neonate from PM- with peripheral blood submicroscopic infection, 1.8% for neonate from healthy controls) (p = 0.02).

**Table 1:** Characteristics of the study population.

Variables	PM chronic infection	PM acute infection	PM- with peripheral blood submicroscopic infection	Healthy control	p values
	(n = 16)	(n = 18)	(n = 43)	(n = 57)	
Age in years, mean ±SD	24.4 ± 4.2	26.3 ± 5.4	27.2 ± 6.9	25.6 ± 5.1	0.31
Parity, mean ±SD	2.1± 1.1	2.2 ± 1.2	2.6 ± 1.5	1.9 ± 1.1	0.06
Maternal hemoglobin levels in g/dL, median [25%-75% IQR]	10.85	11.00	12.35	12.60	P <0.0001
	[9.13, 11.20]	[9.96, 12.60]	[11.40, 13.13]	[11.60, 13.35]	
Baby hemoglobin levels in g/dL, median [25%-75% IQR]	14.80	14.60	15.00	15.30	0.66
	[12.80, 16.00]	[12.63, 16.03]	[13.80, 16.50]	[13.75, 16.40]	
Percentage of maternal anemia (%)	8/16(50,0)	9/18 (50,0)	8/42 (19,0)	7/57 (12,3)	0.0006
Percentage of fetal anemia (%)	3/15 (20.0)	4/18 (22,2)	4/43 (9,3)	1/57 (1,8)	0.02
Baby birth weight, mean ± SD	2,988 ± 406	3,122 ± 409	3,433 ± 473	3,239 ± 498	0.006
Percentage of low birth weight (%)	1/16(6.3)	1/18 (5.6)	1/43 (2.3)	4/57 (7.0)	0.76
Gestational age (weeks), mean ± SD	38 ± 2	39 ± 2	39 ± SD	38 ± SD	0.54
Percentage of preterm delivery (%)	1/16 (6.3)	1/18 (5.6)	4/43 (9.3)	10/57 (17.5)	0.36
Peripheral microscopic parasitemia: trophozoites/μL, median [25%-75% IQR]	26,645	1,691			
	[1,006; 130,409]	[99.0; 20,445]			
Placental intervellous blood microscopic parasitemia: trophozoites/μL, median [25%-75% IQR]	1917	209.5			
	[29.8, 6205]	[0.0, 7613]			
Placental tissue microscopic parasitemia in %, median [25%-75% IQR]	5.8	0.4			
	[0.25, 18.1]	[0.09, 8.1]			
IPTp-SP usage (%)	11/16 (68.8)	15/18 (83.3)	40/43 (93.0)	51/57 (89.5)	0.08
ITNs usage (%)	6/16 (37.5)	14/18 (77.8)	36/43 (83.7)	43/56 (76.8)	0.003

PM: Placenta Malaria; PM- with submicroscopic infection: infection characterised by the positive peripheral blood RT-LAMP result and a negative placental tissue impression smear result; Healthy controls: placental malaria negative women with submicroscopic uninfected peripheral blood.; IPTp-SP: Intermittent preventive treatment with sulphadoxine-pyrimethamine; ITNs: insecticide treated bed net; IQR: interquartile ranges, %: Percentage; Values in brackets represent percentages; SD: Standard deviation. Sample size for ITN is 99 as information about ITN usage was missing for one participant.

The percentage of chronic placental malaria infection (parasite and malaria pigment in the placental tissue) was 47% (16/34) among PM+ women and that of placentas with acute infection (parasite and no malaria pigment in placental tissue) was 53% (18/34). Moreover, the percentage of women who took at least 1 dose of SP as well as those who used ITN was significantly higher in PM-women: 91% (91/100) and 79.8% (79/99) respectively compared to PM+ women: 76.5% (26/34) and 58.8% (20/34) (p = 0.04 and 0.02 respectively).

### Association of CXCL-4, CCL-24, CXCL-13 and CXCL-16 chemokine levels with PM

**Fig 1:**
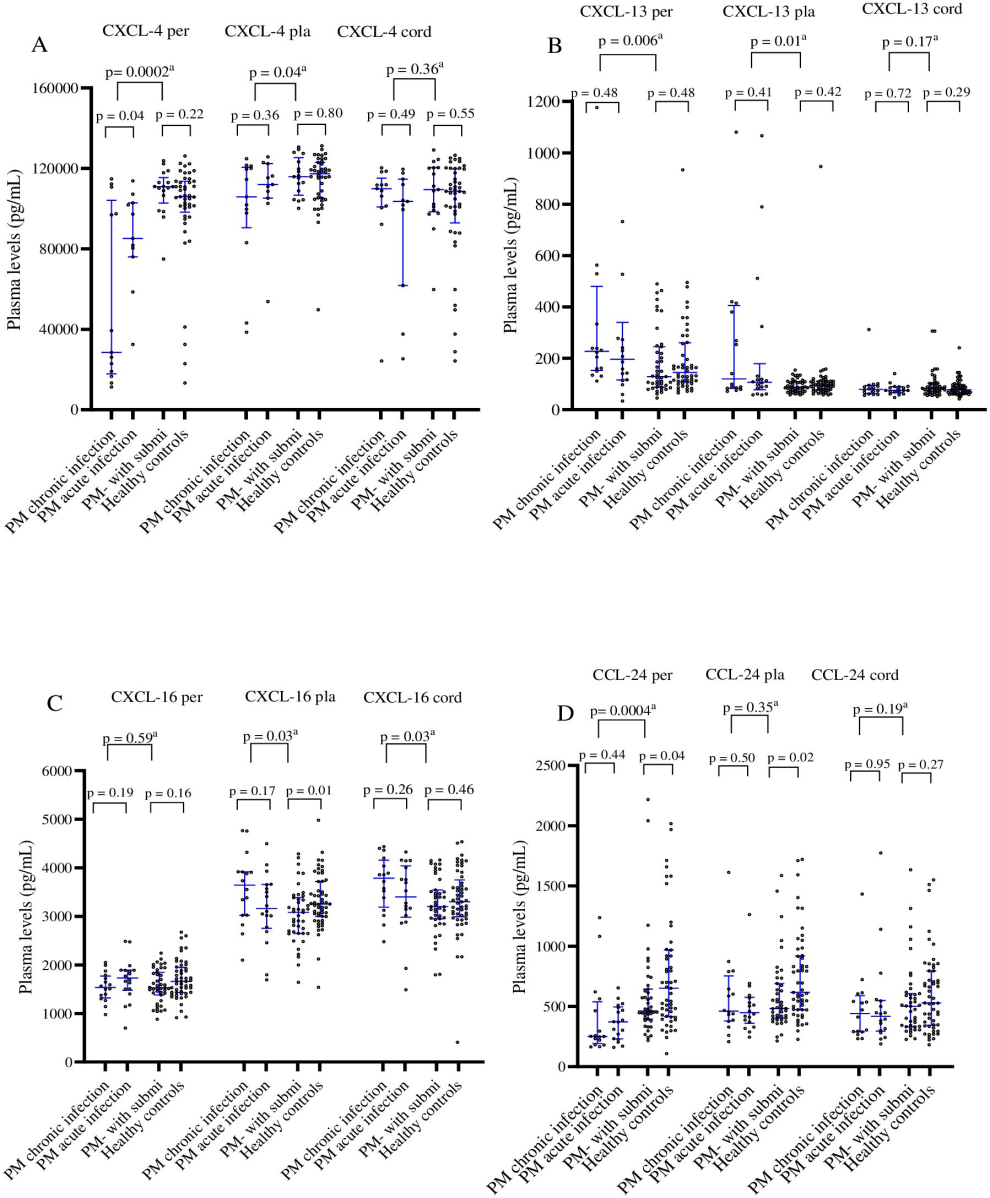
Plasma levels of CXCL-4, CXCL-13, CXCL-16, and CCL-24 chemokines in women at delivery in relation with placental malaria infection. The levels of different chemokines in peripheral (Per), placental (Pla), and cord plasma were compared between different groups, using Man Whitney test. ^a^: comparison between PM+ women and PM-with submicroscopic infection; PM- with submit (PM- with submicroscopic infection): Placental malaria negative women with positive peripheral blood RT-LAMP test; PM+ women: acute and chronic placental malaria positive women; Healthy controls: placental malaria negative women with negative peripheral blood RT-LAMP test.

Plasma levels of CXCL-4, CXCL-13, CXCL-16 and CCL-24 chemokines in the peripheral, placental, and cord blood from PM+ women (PM with chronic and PM with acute infections) and PM- women (PM negative with submicroscopic peripheral blood infection) as well as healthy controls are presented in Fig 1. The levels of CXCL-4 in the peripheral and placenta bloods and CCL-24 in the peripheral blood were significantly lower in PM+ compared to PM- women (median levels in peripheral plasma: CXCL-4: 81, 071 vs. 110, 918 pg/mL, p = 0.0002; CCL-24: 299.9 vs. 462.8 pg/mL, p = 0.0004 and median levels in placental plasma: CXCL-4: 111, 503 vs. 115, 902 pg/mL, p = 0.04). The similar trends but not significant were observed for median level in the placental CCL-24: 457.2 vs. 482.6 pg/mL, (p = 0.35) and in cord plasma: CXCL-4: 107, 535 vs. 109, 430 pg/mL, p = 0.36; CCL-24: 426.1 vs.502.1 pg/mL, p = 0.19).

Conversely, the levels of CXCL-13 were significantly higher in PM+ women compared to PM-women (median levels in the peripheral plasma: 215.2 vs.138.7 pg/mL, p = 0.006, median levels in the placental plasma: 106.6 vs. 86.85 pg/mL, p = 0.01). Concerning CXCL-16, the median levels in the placenta and cord plasma were also significantly higher in PM+ compared to PM- women (median levels in placental plasma: 3,395 vs 3,081 pg/ml, p = 0.03; median levels in cord plasma: 3, 635 vs 3,199 pg/ml, p = 0.03.

### Association of CCL-24 chemokine levels with submicroscopic *P*. *falciparum* infection in women at delivery

The plasma levels of CXCL-4, CXCL-13, CXCL-16 and CCL-24 chemokines in the peripheral, placental, and cord blood of submicroscopic positive women (Placental malaria negative women with submicroscopic infection in peripheral blood) and healthy controls women (Placental malaria negative-women with submicroscopic uninfected peripheral blood) are also presented on Fig 1. The peripheral plasma levels of CXCL-4, CXCL-13 and CXCL-16 chemokines did not differ significantly between submicroscopic positive women and healthy controls women (median levels for CXCL-4: 110, 918 vs 105 892 pg/mL; p = 0.22; for CXCL-13: 128.3 vs 144.1 pg/mL p = 0.48, and for CXCL-16: 1,382 vs 1,433 pg/mL, p = 0.16). A similar trend was observed for CXCL-4 (median: 109,430 vs 92,948 pg/ml, p = 0.55), for CXCL-13 (median:82.91 vs 76.99 pg/ml, p = 0.29), for CXCL-16 (median: 3,199 vs 3,301 pg/ml, p = 0.46) and for CCL-24 (median: 482.6 vs 617.2 pg/ml, p = 0.27) in cord blood.

However, plasma levels of CCL-24 were significantly lower in PM- with submicroscopic positive women compared to the healthy controls (median levels in the peripheral plasma: CCL-24: 462. 8 vs 651. 8 pg/mL; p = 0.040, median levels in the placenta: 482.6 vs 617.2 pg/ml, p = 0.02). A similar trend was observed for placental levels of CXCL-16 (median: 3,081 vs 3,255 pg/ml, p = 0.01).

### Association of CXCL-13 with gravidity in PM+ women

Among PM+ women, the levels of peripheral and placental CXCL-13 chemokine were higher in primigravidae [(N = 12) (272.4 and 323.7 pg/mL respectively)] compared to secundigravidae [(N = 10) (205.3 and 104.6 pg/mL respectively)] and multigravidae women [(N = 12) (158.9 and 89.82 pg/mL respectively)] (p = 0.02 and p = 0.03 respectively). However, the cord plasma levels of these chemokines as well as the levels of CXCL-4, CXCL-16 and CCL-24 in all the blood compartments did not differ significantly between primigravidae, secundigravidae and multigravid women (p> 0.11 for all) (S1 Table).

### Differential association of chemokines with birth weight and maternal or foetal hemoglobin levels

**Fig 2:**
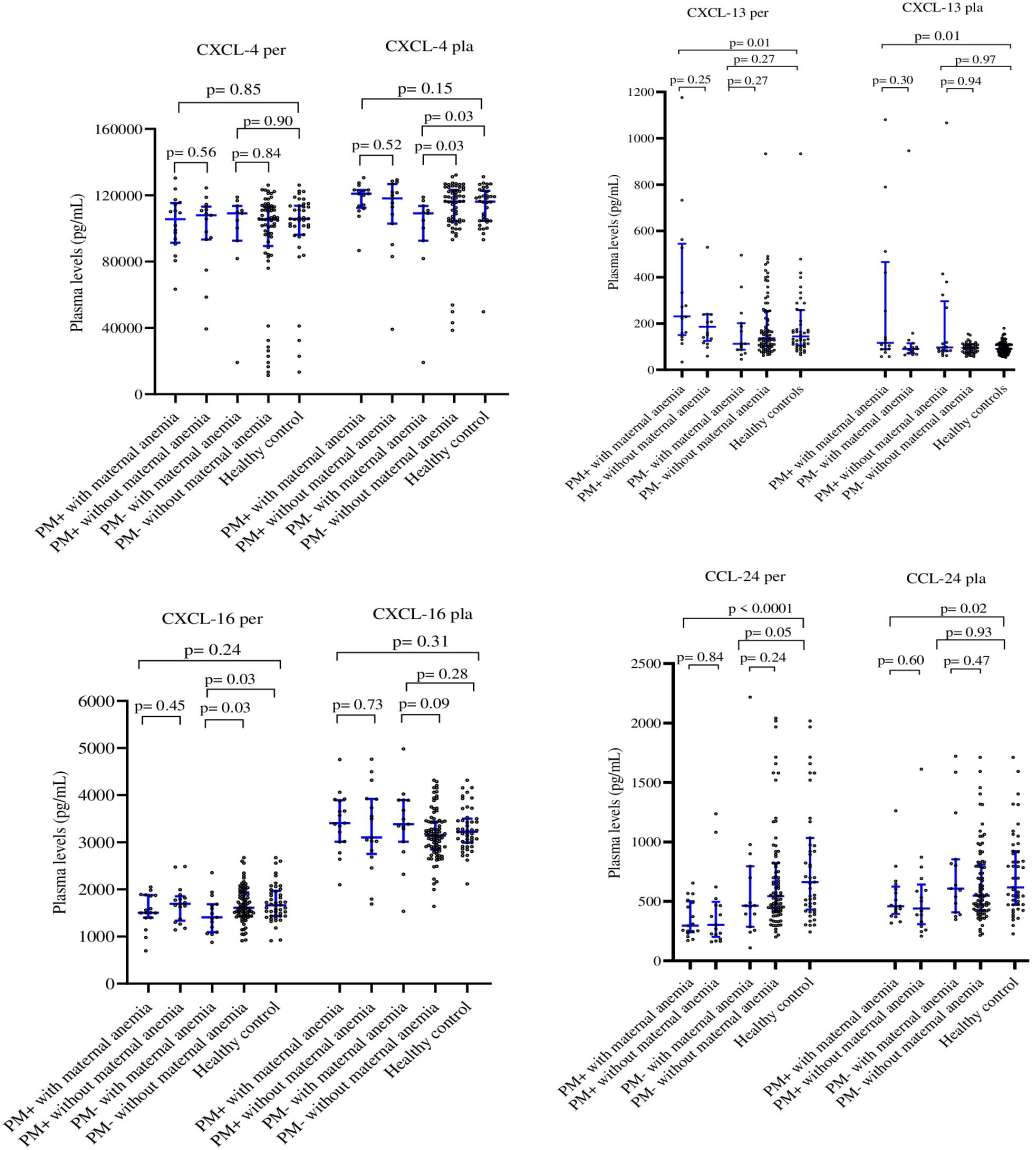
Plasma levels of CXCL-4, CXCL-13, CXCL-16, and CCL-24 chemokines in anemia positive-women and anemia negative-women at delivery. The levels of different chemokines in peripheral (Per), placental (Pla) were compared between anemic and non-anemic women at delivery, using Mann Whitney test. PM-: Placental malaria negative-women; PM+: Placental malaria positive women; healthy controls: placental malaria negative-women with negative peripheral blood RT-LAMP test.

**Fig 3:**
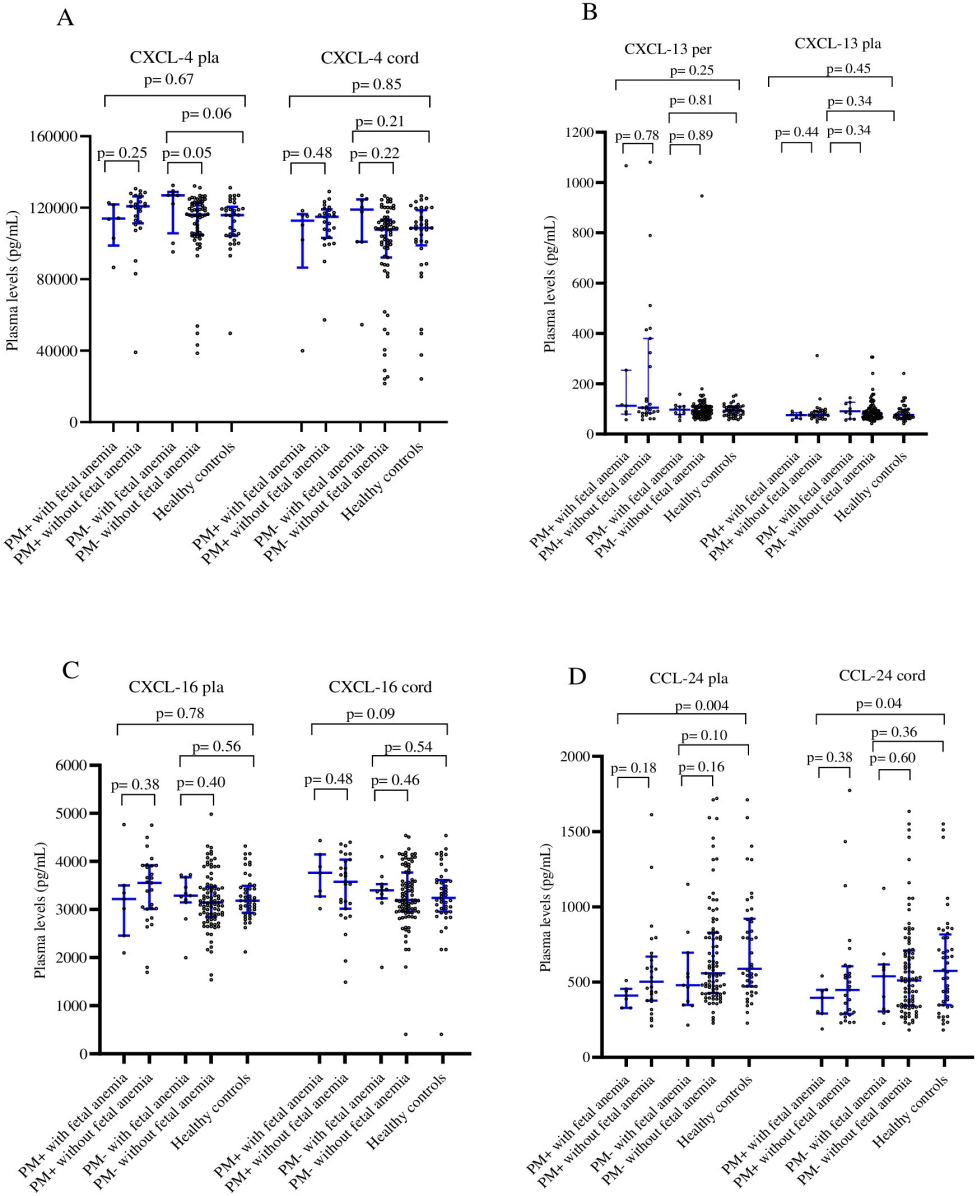
Plasma levels of CXCL-4, CXCL-13, CXCL-16, and CCL-24 chemokines in women at delivery in relation with fetal anemia. The levels of different chemokines in placental (Pla) and cord plasma were compared between the fetal anemia positive-group and fetal anemia negative-women group, using Mann Whitney test. PM-: Placental malaria negative-women; PM+: Placental malaria positive-women; Healthy controls: placental malaria negative women with negative peripheral blood RT-LAMP test.

The data on the relationship between assayed chemokines and birth weight, maternal and neonate hemoglobin levels are presented on the Table 2. The birth weight increased with plasma level of placental and cord CXCL-4 chemokine (p = 0.07 and 0.02, respectively) but decreased with increasing peripheral plasma levels of CXCL-13 chemokine (p = 0.0006). Maternal hemoglobin levels were increased with levels of CXCL-4 and CCL-24 in the peripheral plasma (p = 0.005 and 0.002, respectively), and CCL-24 in the cord plasma (p = 0.008). Similarly, neonate hemoglobin levels were increased with levels of the CCL-24 chemokine in peripheral, placental and cord plasma (p = 0.022, 0.030 and 0.019 respectively), and with CXCL-4 in peripheral plasma (p = 0.003) (Table 2). As shown in Fig 2, only CXCL-13 and CCL-24 in both peripheral (p = 0.01 and <0.0001, respectively) and placental (p = 0.01 and p = 0.02, respectively) plasma were associated significantly with maternal anemia in PM+ women when compared to the healthy controls. Interestingly, the increase in CXCL-13 was accompanied by decrease in CCL-24, suggesting opposing roles for these two chemokines in PM-associated anemia. In contrast, Fig 3 shows that, only CCL-24 in both placental and cord plasma was associated significantly (p = 0.004 and 0.04, respectively) with fetal anemia in PM+ women.

**Table 2:** Plasma levels of chemokines in relation to birth weight, maternal hemoglobin and foetal haemoglobin levels.

Variables	**[CXCL-4 per] ≤ Q/4**	**Q/4 < [CXCL-4 per] ≤ 3Q/4**	**[CXCL-4 per] > 3Q/4**	**p value**
Birth weight	3502 ± 441	3284 ± 562	3331 ± 544	0.99
Maternal Hb	11.80 [9.50; 12.70]	12.75 [11.80; 13.40]	12.70 [11.75; 13.70]	0.005
Cord Hb	14.45 [12.95; 15.40]	15.30 [14.58; 16.33]	15.75 [14.30; 16.98]	0.003
	**[CXCL-4 pla] ≤ Q/4**	**Q/4 < [CXCL-4 pla] ≤ 3Q/4**	**[CXCL-4 pla] > 3Q/4**	**p value**
Birth weight	3213 ± 558	3242 ± 499	3495 ± 505	0.07
Maternal Hb	12.90 [11.20; 13.30]	12.55 [11.58; 13.40]	12.50 [11.55; 13.55]	0.92
Cord Hb	15.10 [14.45; 16.50]	15.05 [14.00; 16.30]	15.50 [14.50; 16.40]	0.67
	**[CXCL-4 cord] ≤ Q/4**	**Q/4 <[CXCL-4 cord] ≤ 3Q/4**	**[CXCL-4 cord] > 3Q/4**	**p value**
Birth weight	3070 ± 465	3339 ± 580	3452 ± 387	0.02
Maternal Hb	12.60 [11.80; 13.23]	12.50[11.20; 13.55]	12.50 [11.60; 13.50]	0.94
Cord Hb	14.95 [14.48; 16.23]	15.20 [14.00; 16.78]	15.05 [13.90; 16.38]	0.91
	**[CXCL-13 per] ≤ Q/4**	**Q/4 < [CXCL-13 per] ≤ 3Q/4**	**[CXCL-13 per] > 3Q/4**	**p value**
Birth weight	3523 ± 419	3257 ± 524	3194 ± 476	0.0006
Maternal Hb	12.10 [11.40; 12.60]	12.40 [11.05; 13.30]	12.00 [10.35; 13.05]	0.69
Cord Hb	14.90 [13.90; 15.80]	14.90 [13.75; 16.35]	14.70 [12.80; 14.70]	0.87
	**[CXCL-13pla] ≤ Q/4**	**Q/4 < [CXCL-13 pla] ≤ 3Q/4**	**[CXCL-13 pla] > 3Q/4**	**p value**
Birth weight	3312 ± 468	3153 ± 441	3176 ± 497	0.62
Maternal Hb	11.90 [11.10; 12.53]	12.65 [11.35; 13.30]	11.70 [9.90; 12.70]	0.007
Cord Hb	14.80 [13.75; 15.93]	14.85 [13.73; 16.35]	15.80 [13.10; 16.70]	0.62
	**[CXCL-13cord]≤ Q/4**	**Q/4< [CXCL-13 cord] ≤ 3Q/4**	**[CXCL-13 cord]> 3Q/4**	**p value**
Birth weight	3282± 368	3240 ± 526	3259 ± 519	0.92
Maternal Hb	11.90 [10.60; 12.80]	11.90 [10.70; 13.20]	12.35 [11.63; 13.28]	0.14
Cord Hb	14.70 [13.48; 15.55]	14.90 [13.70; 16.30]	15.85 [14.03; 17.33]	0.07
	**[CXCL-16per] ≤ Q/4**	**Q/4 < [CXCL-16per] ≤ 3Q/4**	**[CXCL-16per] > 3Q/4**	**p value**
Birth weight	3145 ± 523	3262 ± 397	3350 ± 592	0.23
Maternal Hb	11.50 [9.8; 12.80]	12.25 [11.20; 13.03]	12.55 [11.55; 13.43]	0.03
Cord Hb	14.90 [13.80; 16.35]	14.80 [13.68; 16.05]	15.75 [13.98; 16.53]	0.52
	**[CXCL-16 pla] ≤ Q/4**	**Q/4 < [CXCL-16 pla] ≤ 3Q/4**	**[CXCL-16 pla] > 3Q/4**	**p value**
Birth weight	3227 ± 479	3287 ± 475	3220 ± 525	0.28
Maternal Hb	12.50 [11.50; 13.20]	12.10 [11.20; 13.20]	11.60 [9.9; 12.90]	0.19
Cord Hb	15.20 [13.75; 16.90]	14.85 [13.70; 16.38]	14.85 [13.83; 15.88]	0.48
	**[CXCL-16 cord] ≤ Q/4**	**Q/4< [CXCL-16 cord] ≤ 3Q/4**	**[CXCL-16 cord]> 3Q/4**	**p value**
Birth weight	3224 ± 431	3333 ± 513	3120 ± 460	0.11
Maternal Hb	12.60 [11.85; 13.20]	12.10 [10.93; 13.28]	11.55 [10.28; 12.68]	0.05
Cord Hb	15.80 [14.23; 17.08]	14.80 [12.80; 16.10]	14.80 [13.83; 15.98]	0.05
	**[CCL-24 per] ≤ Q/4**	**Q/4 < [CCL-24 per] ≤ 3Q/4**	**[CCL-24 per] > 3Q/4**	**p value**
Birth weight	3157 ± 469	3310 ± 415	3247 ± 619	0.33
Maternal Hb	11.20 [9.92; 12.28]	12.60 [11.40; 13.20]	12.05 [11.53; 13.20]	0.002
Cord Hb	14.10 [12.70; 15.35]	15.30 [13.70; 16.60]	16.00 [14.45; 16.50]	0.022
	**[CCL-24 pla] ≤ Q/4**	**Q/4 < [CCL-24 pla] ≤ 3Q/4**	**[CCL-24 pla] > 3Q/4**	**p value**
Birth weight	3386± 509	3194 ± 399	3252 ± 602	0.17
Maternal Hb	11.85 [10.88;12.85]	12.10 [11.15; 12.93]	12.60 [10.85; 13.35]	0.45
Cord Hb	14.65 [12.73; 15.83]	14.75 [13.68; 16.28]	16.00 [14.50; 16.50]	0.030
	**[CCL-24 cord]≤ Q/4**	**Q/4< [CCL-24 cord] ≤ 3Q/4**	**[CCL-24 cord] > 3Q/4**	**p value**
Birth weight	3300 [2900; 3600]	3300 [3100; 3500]	2975 [2613; 3475]	0.08
Maternal Hb	11.50 [9.55; 12.50]	12.30 [11.20; 13.10]	13.00 [11.30; 13.75]	0.008
Cord Hb	14.40 [12.88; 15.65]	14.90 [13.65; 16.25]	16.10 [14.48; 16.55]	0.019

Hb: hemoglobin level, Q/4: 25^th^ percentiles, 3Q/4: 75^th^ percentiles, Per: peripheral plasma, Pla: placental plasma. Birth weight was expressed as mean with standard deviation.

## Discussion

The main findings of this study were: 1) the association of PM with enhanced levels of CXCL-13, CXCL-16 and low levels of CXCL-4 and CCL-24 in peripheral and placental plasma; 2) the association of submicroscopic *P*. *falciparum* parasitemia with decreased levels of CCL-24 in both peripheral and placental plasma. Moreover, maternal hemoglobin level in the present study increased with peripheral plasma levels of CXCL-4 while birth weight was lower for babies born from women with elevated levels of peripheral CXCL-13 but low plasma levels of cord CXCL-4 and CCL-24. These findings suggest that low levels of CXCL-4 and CCL-24 coupled with high plasma levels of CXCL-13 might represent the signature of PM and associated outcomes including maternal anemia and low birth weight. These findings have implications in the development of complementary diagnostic tools for PM diagnosis and prediction of placental malaria outcomes. Several studies have highlighted the involvement of cytokines and chemokines in the pathophysiology of the disease, but this study is the first to report the involvement of CXCL-4 and CCL-24 in PM. In a previous study, Wilson *et al* in 2011 [25] reported high expression of CXCL-4 in children with cerebral malaria, which contrast with the reduced levels of the chemokine found in PM+ women in the present study. This discrepancy might be explained by differences in the immuno-pathogenesis mechanisms of placental and cerebral malaria that include the differential expression of certain cytokines including CXCL4 and CCL24. According to previous studies, CXCL-4 binds to the erythrocyte Duffy antigen receptor, promoting lysis of the latter [26]. Moreover, this chemokine has been shown to exhibit both pro and anti-inflammatory effects as its level was significantly low in patients with acute hepatitis, and its absence was associated with severe liver damage [27]. CXCL-4 also has been shown to promote hematopoiesis by retaining progenitor cells [28], and this is consistent with the positive association observed in this study between plasma levels of CXCL-4 and maternal hemoglobin level.

Concerning CCL-24, pregnant women trophoblasts have been shown as one of main sources of this chemokine which binds to CCR3 receptors in decidual stromal cells [29]. In addition, the production of the progesterone, estrogen and human chorionic gonadotropin hormones is associated with upregulation of CCL-24 [29]. Thus, the low levels of CCL-24 found in this study in PM positive women could be due to the decrease in progesterone expression [30] and an alteration of the trophoblast [31] having been shown to be associated with PM. Placentas from women who were infected with *P*. *falciparum* during early pregnancy showed signs of damages such as reduced transport villi, increased syncytial knotting and increased placental lesions [32–34], while active infection at delivery was associated with reduced villous area and vascularity, increased basal membrane thickening, syncytial damage, increased syncytial knotting and fibrinoid necrosis [32, 35]. The enhanced plasma levels of CXCL-13 chemokine found in the present study in PM + women compared to PM-women is in agreement with previous studies [3, 4].

Submicroscopic parasitaemia is often associated with altered immune profile [36]. For the first time, we show in this study in relation with submicroscopic infection that the plasma concentration of CCL-24 in the peripheral and placental plasma was significantly higher in healthy controls compared to submicroscopic positive women. Only placental plasma concentration of CXCL-16 was higher in healthy controls. These results suggest that submicroscopic infection is also associated with decrease of peripheral and placental plasma concentration of CCL-24 and placental concentration of CXCL-16.

PM is known to cause maternal anemia, fetal anemia and low birth weight. Thus, the positive association observed between the levels of CXCL-4, CXCL-16, CCL-24 (in the peripheral, placental or cord blood depending on chemokine) and maternal or fetal Hb and between birth weight and CXCL-4 chemokine in the cord plasma, suggests the protective role of these chemokines against PM. In addition, the negative association observed between CXCL-13 levels in the peripheral plasma and birth weight as well as between the placental levels of this chemokine and the maternal hemoglobin level suggest its association with PM poor pregnancy outcomes.

The positive association found in the present study between CXCL-13 and decreasing birth weight is in line with previous findings by others [3, 4]. Indeed, CXCL-13 is produced by macrophage to activate the inflammatory T cells responses that might contribute to increased inflammatory cell infiltration and fibrinoid deposits and oxidative stress in the placenta [8, 37]. Increased oxidative insults may trigger apoptosis in human placenta [38, 39] and in mouse model of PM [40]. Collectively, the accumulation of fibrin in the intervellous spaces coupled with dysregulation of angiogenesis in the placenta can result in inadequate perfusion to placenta, causing necrosis [41] that may lead to growth restriction and low birth weight. Gravidity had no influence on the plasma levels of different assayed chemokines. The observed decrease in CXCL-13 in the peripheral blood with increasing gravidity among PM+ group can be explained on the basis of differences in parasitaemia that have been shown to be higher in paucigravid women compared to multigravid women.

## Conclusion

These results suggest that conversely to high plasma levels of CXCL-13 and for a lesser extend CXCL-16, confirmed to be associated to *P*. *falciparum* PM, high plasma levels of CXCL-4 and CCL-24 chemokines might be associated with low risk of the disease in pregnant women. These findings are relevant for the identification of correlates of protection against PM and targets that can be used for the development of complementary diagnostic tools.

## Supporting information

S1 Table Comparison of chemokines plasma levels between primigravid, secundigravid, and multigravid women at delivery. (DOCX)

S1 Data Data base. (XLSX)
