# Supplementary material for: Chemokine modulation in microscopic and submicroscopic Plasmodium falciparum malaria infection in women at delivery in Yaoundé, Cameroon
Source: PLoS One. 2023 Jan 23;18(1):e0280615. doi: 10.1371/journal.pone.0280615 (PMC9870109; doi:10.1371/journal.pone.0280615)
Supplement: S1 Table — (DOCX) [file pone.0280615.s001.docx]

S1 Table: Comparison of chemokines plasma levels between primigravid, secundigravid, and multigravid women at delivery.

| Chemokines concentration in pg/mL | Placental malaria | | | |  |  | Healthy controls | | | |
| --- | --- | --- | --- | --- | --- | --- | --- | --- | --- | --- |
|  | Primigravidae  N= 11 | Secundigravidae  N= 9 | Multigravidae  N= 14 | P-Value |  |  | Primigravidae  N=24 | Secundigravidae  N=16 | Multigravidae  N=17 | P-value |
| CXCL-4 Per | 24,552  [17,214 ; 93,179] | 97,255  [39,337 ; 107,203] | 85,129  [45,519; 111,507] | 0.10 |  |  | 104,852  [89,087; 110,179] | 105,579  [101,015 ; 114,675] | 109,487  [94,289; 116,894] | 0.40 |
| CXCL-4 Pla | 103,828  [45,810 ; 113,869] | 116,267  [99,710 ; 122,362] | 111,001  [103,886 ;122,995] | 0.14 |  |  | 118,159  [106,622;121,241] | 122,285  [119,314 ; 125,935] | 118,645  [104,220;125,820] | 0.12 |
| CXCL-4 Cord | 104,852  [100,618; 111,781] | 104,112  [92,211 ; 114,704] | 109,903  [80,607; 118,133] | 0.83 |  |  | 104,063  [68,030; 109,468] | 109,442  [99,510 ; 119,530] | 112,338  [101,055;119,856] | 0.13 |
| CXCL-13 Per | 272.4  [207.3 ; 1510] | 205.3  [121.69; 228.0] | 158.9  [124.9 ; 233.4] | **0.02** |  |  | 167.8  [111.0 ; 304.5] | 129.3  [85.23 ; 172.0] | 142.1  [88.83 ; 309.4] | 0.35 |
| CXCL-13 Pla | 323.7  [108.6 ; 1067] | 104.6  [75.01 ; 417.5] | 89.82  [71.56; 121.4] | **0.03** |  |  | 94.75  [78.96 ; 113.5] | 98.70  [77.48 ; 108.6] | 85.87  [66.62 ; 98.20] | 0.26 |
| CXCL-13 Cord | 88.83  [71.06 ; 98.70] | 75.01  [64.16 ; 80.93] | 72.05  [58.73 ; 90.31] | 0.11 |  |  | 88.83  [61.69 ; 106.6] | 78.47  [61.19 ; 91.30] | 72.05  [65.64 ; 84.14] | 0.68 |
| CXCL-16 Per | 1630  [1506 ; 1994] | 1402  [1320 ; 1684] | 1707  [1411 ; 1832] | 0.19 |  |  | 1589  [1394 ; 1967] | 1727  [1433 ; 2047] | 1632  [1462 ; 1873] | 0.79 |
| CXCL-16 Pla | 3568  [2458 ; 3916] | 3384  [3018 ; 4189] | 3077  [2812 ; 3770] | 0.67 |  |  | 3265  [3058 ; 3821] | 3183  [2861 ; 3461] | 3555  [2905 ; 3901] | 0.59 |
| CXCL-16 Cord | 3762  [2823 ; 4144] | 3526  [3018 ; 4300] | 3453  [3142 ; 3917] | 0.73 |  |  | 3193  [2890 ; 3751] | 3240  [2962 ; 3527] | 3559  [3126 ; 4138] | 0.15 |
| CCL-24 Per | 371.7  [247.4 ; 490.9] | 250.7  [176.6 ; 458.4] | 303.3  [230.8 ; 534.4] | 0.45 |  |  | 700.4  [164.0 ; 1344] | 621.1  [421.2 ; 1019] | 548.7  [397.6 ; 794.4] | 0.60 |
| CCL-24 Pla | 386.3  [317.4 ; 531.3] | 511.3  [425.1 ; 615.3] | 478.8  [407.9 ; 716.3] | 0.16 |  |  | 793.2  [549.6 ; 1069] | 598.6  [473.4 ; 838.2] | 473.7  [395.5 ; 775.2] | 0.08 |
| CCL-24 Cord | 359.5  [267.6 ; 471.1] | 395.7  [261.7 ; 609.1] | 514.4  [301.9 ; 548.8] | 0.50 |  |  | 671.2  [392.5 ; 839.6] | 488.8  [333.8 ; 709.6] | 392.6  [275.6 ; 659.7] | 0.11 |

Per: Peripheral plasma, Pla: placental plasma, values are presented as medians with interquartile ranges (25 and 75 percentiles). Healthy controls: placental malaria negative women with submicroscopic uninfected peripheral blood.
